# Saline Retention and Permeability of Nanofiltration Membranes Versus Resistance and Capacitance as Obtained from Impedance Spectroscopy under a Concentration Gradient

**DOI:** 10.3390/membranes13060608

**Published:** 2023-06-18

**Authors:** Miguel-Ángel Pérez, Silvia Gallego, Laura Palacio, Antonio Hernández, Pedro Prádanos, Francisco Javier Carmona

**Affiliations:** Grupo de Superficies y Materiales Porosos, Departamento de Física Aplicada, Facultad de Ciencias, Universidad de Valladolid, Paseo de Belén 7, 47011 Valladolid, Spain; miguelangel.perez22@estudiantes.uva.es (M.-Á.P.); silviateresa.gallego@uva.es (S.G.); laura.palacio@uva.es (L.P.); antonio.hernandez@uva.es (A.H.); ppradanos@uva.es (P.P.)

**Keywords:** nanofiltration, retention, permeability, zeta potential, impedance spectroscopy

## Abstract

Impedance spectroscopy has been widely used for the study of the electrical properties of membranes for their characterization. The most common use of this technique is the measure of the conductivity of different electrolyte solutions to study the behavior and movement of electrically charged particles inside the pores of membranes. The objective of this investigation was to observe if there is a relation present between the retention that a nanofiltration membrane possesses to certain electrolytic solutions (NaCl, KCl, MgCl_2_, CaCl_2_, and Na_2_SO_4_) and the parameters that are obtained through IS measurements of the active layer of the membrane. To achieve our objective, different characterization techniques were performed to obtain the permeability, retention, and zeta potential values of a Desal-HL nanofiltration membrane. Impedance spectroscopy measurements were performed when a gradient concentration was present between both sides of the membrane to study the variation that the electrical parameters had with the time evolution.

## 1. Introduction

Nanofiltration (NF) has been proven to be a good alternative to reverse osmosis (RO) for poor brackish waters that have divalent ions or solutes with low molecular weight (~300 Da), due to the low pressures required [1,2] and the high permeate fluxes obtained. When NF is applied for ion retention, its performance for that purpose depends strongly on the charge of the membrane [3,4,5] and their interaction with the charges of the ions. The effects of the charges of the membrane surface appear mainly in the case of NF membranes through two processes: the ionization of functional groups [6,7,8] and the absorption of charged molecules [7,9]. The many applications of nanofiltration make it highly desirable to fully characterize NF membranes so that the retention phenomena can be understood and a better filtration process can be achieved. When characterizing a membrane, the most studied properties are those related to the properties of retention that the membrane presents to the passing of certain molecules. Some of the most measured parameters that give an account of the transport of molecules through a NF membrane are retention, permeability, and zeta potential. The latter is quite relevant as it has been proven to be strongly correlated with the value of retention [10,11]. The measurement of these parameters has been commonly accomplished by different authors and exposed in multiple articles [12,13,14] for different types of membranes.

Electrochemical impedance spectroscopy (EIS), or simply impedance spectroscopy (IS), is a relatively modern characterization technique for many materials and has been used in many scientific and technological areas [15,16]. In the particular case of membranes or porous materials, EIS [17,18] has been used by different authors for the characterization of the behavior of some electrolyte solutions inside the pores of NF membranes, which can give a great amount of information about the electrical properties of the system and the movement of salts inside the pores [14,19,20,21]. In 2001, Fievet et al. [22] used EIS to study, both experimentally and theoretically, the dependance that exists between the conductivity of different salts inside the pores of an ultrafiltration (UF) membrane and the value of zeta potential that the membrane presented when it was immersed into such solutions. In that study, they showed how the relation between conductivity and zeta potential could define a possible technique of zeta potential measurement which could be applied even in the case of NF membranes. This latest application of EIS would suppose to remove the disadvantages that presents the flux gradient method with small pores, as it can commonly lead to serious problems at low concentrations.

Here, we will study the dependence and possible correlation between the electrical parameters that are normally used in EIS to characterize a NF membrane when the membrane is immersed in different electrolyte solutions (NaCl, KCl, MgCl_2_, CaCl_2_, and Na_2_SO_4_). This study will lead us to correlate the EIS data with important parameters for the description of membrane performance such as permeability, zeta potential, or retention. EIS measurements were performed in the presence of a concentration gradient between both sides of the membrane, forcing a flux of ions through until equilibrium was reached. This experimental setup was previously compared with the usual EIS experimental setup, where a solution with a fixed concentration is pumped to both sides of the membrane until equilibrium is reached. The gradient technique allows a detailed analysis of the characteristics of the active layer and their evolution with time, therefore approaching the real nanofiltration procedure. EIS measurements were taken for the membrane + solution system and followed with time so as to observe the time evolution of the electrical properties of the system, especially those related to the membrane’s active layer, and to see if there exists a relationship between the calculated electrical parameters and the retention values measured through conventional techniques. This relationship could imply that EIS could be used in the study of membranes for a deep understanding of the behavior of electrolytic solutions inside the pores.

## 2. Materials and Methods

In this work, experimental measurements of impedance spectroscopy, saline permeability, and tangential streaming potential were carried out for the characterization of a nanofiltration membrane using five different saline solutions.

### 2.1. Membrane

The membrane used in this work was the DESAL-HL, which is a plane nanofiltration membrane manufactured by GE-Osmonics (Minnetonka, MN, USA). It is a polyamide membrane with a molecular weight cutoff (MWCO) between 150 and 300 g/mol [23] and that can be used in media with a pH ranging between 3 and 9 and at a maximum temperature of 50 °C, according to the manufacturer. It has a previously evaluated mean pore radius of 0.47 nm [19].

### 2.2. Electrolytic Solutions

The salts used in this experiment were NaCl and KCl (electrolyte type 1:1), MgCl_2_ and CaCl_2_ (type 1:2), and Na_2_SO_4_ (type 2:1). The salts MgCl_2_, CaCl_2_, and Na_2_SO_4_ were supplied by Panreac (ITW, Glenview, IL, USA), KCl by VMR Chemicals (Radnor, PA, USA), and NaCl by Merck (Darmstadt, Hesse, Germany).

Saline aqueous solutions were prepared with ion exchange and reverse-osmosis-treated demineralized water using a miliQ (Millipore, Subsidiary of Merck KGaA, Billerica, MA, USA).

### 2.3. Electrochemical Impedance Spectroscopy

EIS measurements were performed at room temperature using a Solartron 1260 impedance/gain-phase analyzer (AMETEK, Berwyn, Pennsylvania) in an in-circuit impedance measurement scheme. Each of the spectral measurements was conducted with an AC signal of 50 mV of amplitude and a range of decreasing frequencies between 0.1 Hz and 1 MHz. Ten points per decade were taken so Nyquist plots of the impedance behavior had enough resolution to distinguish the behavior at both layers of the membrane. Samples of our system under test, a Desal-HL membrane, were placed between two matching methacrylate semicells with an area of 10.18 cm^2^ open to the flux of the electrolyte solution; this same experimental setup was previously described by Montalvillo et al. [24]. In our case, two Cl^−^-selective electrodes were placed close to the membrane to perform EIS measurements. To reduce the signal error, a typical four-wire impedance measurement was designed to eliminate the inherent impedance of the lead wire.

**Figure 1:**
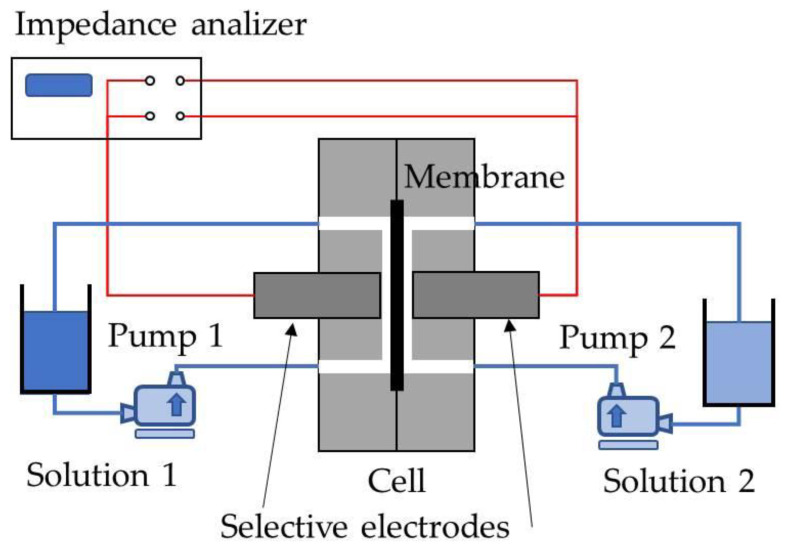
Impedance spectroscopy experimental setup for the measurement of electrical parameters of our NF membrane for the different electrolyte solutions.

Two recipients, placed at both sides of the membrane, were filled with 0.56 L of deionized water in which we added a highly concentrated electrolyte solution, until the desired concentrations were reached. The concentrations were designed as high concentration ($c_{\mathrm{high}}$) on the side of the same name, and low concentration ($c_{\mathrm{low}}$) for the concentration on the other side. These were intended to take initial values of 10^−3^ mol/L and 10^−5^ mol/L, respectively, in the case of monovalent salts and 5×10^−4^ mol/L and 5×10^−6^ mol/L for the divalent salts. These concentration ranges correspond to the electrodes’ range of working, and they are also the appropriate range for the study of zeta potential. The saline solutions of the five different salts prepared in this way contained the same charge concentration in all cases (NaCl, KCl, CaCl_2_, MgCl_2_, CaCl_2_, and Na_2_SO_4_). The first four salts were chosen to have Cl^−^ as a common anion in order to analyze the cation effect: two with one positive charge and two with two positive charges. In each pair of salts with the same elemental charge, the size and diffusivity were different. The Na_2_SO_4_ salt was chosen for the sake of analyzing the effect of the anion when it is more voluminous and more negative. The real concentrations, slightly different from the desired ones, were measured experimentally as indicated in Section 2.4. The membrane was previously submerged for several days in the low-concentration solution to precondition it. In order to reduce the concentration polarization, it is very important to keep both sides properly stirred; this was ensured by recirculating the solution with a pump with a tangential flux of 0.6 L/min on each side of the membrane. A scheme of the experimental device is shown in Figure 1.

The measurements of the impedance spectrum were automatically recorded with a computer. The adjustment of the experimental results to the corresponding equivalent electrical circuit to obtain the electrical parameters of this circuit was carried out using the commercial Zview software (AMETEK, Berwyn, PA, USA). This fit procedure used the ‘Calc-Modulus’ data weight type; each data point’s weight was normalized by its magnitude.

### 2.4. Retention and Permeability

Salt retention and permeability were measured simultaneously and using the same cell as for the impedance spectroscopy measurements. When performing the retention and permeability measurements, the concentration was measured using a HI 5522 conductivity meter (Hanna Instruments, Padua, Italy). Details of the experimental device can be found in a previous work [14].

The process was developed over 30 h, during which, in four representative moments, the EIS measurements were made. This was at time points *t* = 0 h, *t* = 6 h, *t* = 23 h, and *t* = 29 h. The first two of them we will call “initial times”, and the last two we will call “final times”, of the retention and permeability process.

### 2.5. Tangential Streaming Potential and Zeta Potential

With the aim of evaluating the zeta potential, tangential streaming potential measurements were carried out [25,26,27]. The experimental setup used for these measurements consisted of a membrane holder where the active faces of two sheets of the membrane were placed face to face, leaving a single channel through which the solution was circulated. This holder did not allow permeation through the membrane.

**Figure 2:**
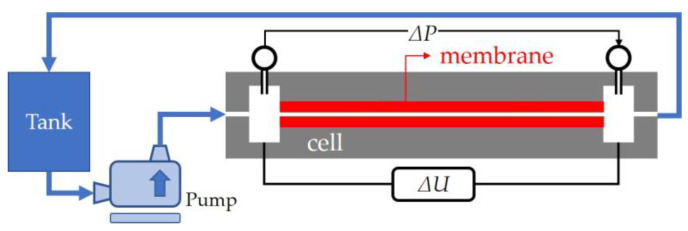
Tangential streaming potential experimental setup for the experimental measurement of zeta potential values for the different electrolyte solutions.

The experimental procedure consisted of applying different pressure gradients and measuring the produced electrical potential. The solution flowed tangentially to the membrane surfaces thanks to the pressure gradient generated with a pump. The electrical potential was measured by using a high-impedance (greater than 10^10^ Ω) Hewlett-Packard voltmeter (HP3456A) with an accuracy of 1 µV. The system used for the experimental obtention was the same setup used by Silva et al. [28,29] and shown in Figure 2. The measurements were made at the natural pH of the salt solution in deionized water.

## 3. Results and Discussion

### 3.1. Retention and Permeability

Saline permeability (*P*) is a characteristic parameter relating the flux (*j*) of a saline solution through a membrane, when a concentration difference ($c_{\mathrm{high}}-c_{\mathrm{low}}$) exists between both faces of the membrane. According to Fick’s law,

(1) $$j=P\left(c_{\mathrm{high}}-c_{\mathrm{low}}\right)=\frac{V_{\mathrm{low}}}{A}\;\frac{dc_{\mathrm{low}}\left(t\right)}{dt}=-\frac{V_{\mathrm{high}}}{A}\;\frac{dc_{\mathrm{high}}\left(t\right)}{dt}$$

In the experimental arrangement used here, the volumes of the high-concentration side and the low-concentration sides are equal, $V_{\mathrm{low}}=V_{\mathrm{high}}\overset{\mathrm{def}}{=}$ *V*, and the exchange of salt occurs through the membrane area open to the flux (*A*). Therefore the variations of high and low concentrations will be the same although of opposite sign $dc_{\mathrm{low}}\left(t\right)/dt=-dc_{\mathrm{high}}\left(t\right)/dt$*,* being its sum constant at all times, $c_{\mathrm{high}}\left(t\right)+c_{\mathrm{low}}\left(t\right)=c_{\mathrm{high}}\left(0\right)+c_{\mathrm{low}}\left(0\right)$. Then, both the concentrations evolve to the equilibrium value, so that for a long enough time (*t*→∞), it could be considered that the concentrations of both solutions will equal the value of the initial average, $c_{\mathrm{high}}\left(\infty \right)=c_{\mathrm{low}}\left(\infty \right)=\left(c_{\mathrm{high}}\left(0\right)+c_{\mathrm{low}}\left(0\right)\right)/2\overset{\mathrm{def}}{=}c\left(\infty \right)$. With all this in mind, the concentration on the low-concentration side will evolve over time according to Equation (2), as used previously by Diaz et al. [14]:

(2) $$f\left(c_{\mathrm{low}}\left(t\right)\right)\overset{\mathrm{def}}{=}\ln\left[\frac{c_{\mathrm{low}}\left(t\right)-c_{\mathrm{low}}\left(\infty \right)}{c_{\mathrm{low}}\left(0\right)-c_{\mathrm{low}}\left(\infty \right)}\right]=-\left(\frac{2AP}{V}\right)t$$

**Figure 3:**
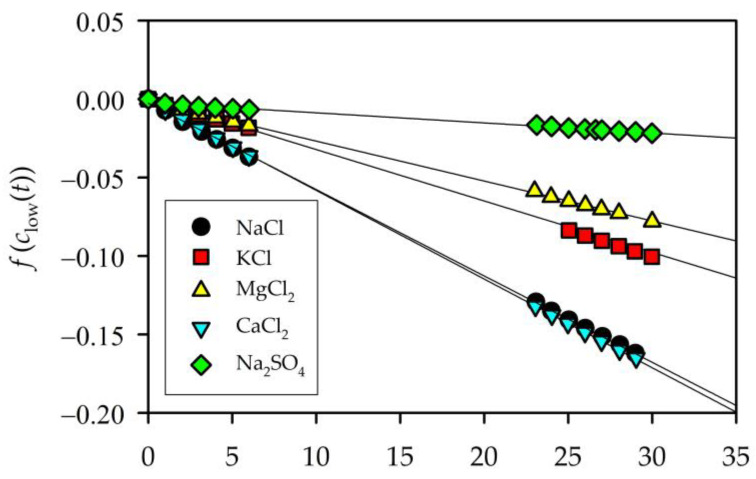
Linear fits for the time evolution of $f\left(c_{\mathrm{low}}\left(t\right)\right)$ defined in Equation (2).

The linear fits of $f\left(c_{\mathrm{low}}\left(t\right)\right)$ against time allow us to know the permeabilities for each solution (Figure 3). For all the electrolytes studied in this work, the corresponding fit showed a strong linear relationship, indicating that the equation used for calculus is applicable. The permeability values are shown in Table 1 for the electrolyte solutions used in this work.

The observed retention coefficient presented by the membrane, *R*_obs_, is calculated in this case as:

(3) $$R_{\mathrm{obs}}=1-\frac{c_{\mathrm{low}}}{c_{\mathrm{high}}}$$

According to the experimental procedure described above, the concentrations on each side of the membrane should evolve according to the following expressions, obtained via the integration of Equation (2) and taking into account the limits of integration indicated.

(4) $$\begin{array}{c} c_{\mathrm{high}}\left(t\right)=c_{\mathrm{high}}\left(0\right)-0.5\left(c_{\mathrm{high}}\left(0\right)-c_{\mathrm{low}}\left(0\right)\right)\left[1-\exp\left\{-\frac{2AP}{V}t\right\}\right]\\ c_{\mathrm{low}}\left(t\right)=c_{\mathrm{low}}\left(0\right)+0.5\left(c_{\mathrm{high}}\left(0\right)-c_{\mathrm{low}}\left(0\right)\right)\left[1-\exp\left\{-\frac{2AP}{V}t\right\}\right] \end{array}$$

Additionally, indeed, this is how they behave experimentally. In this way, retention would evolve according to the relationship obtained by substituting Equation (4) in Equation (3):

(5) $$R_{\mathrm{obs}\;}=\frac{\left(c_{\mathrm{high}}\left(0\right)-c_{\mathrm{low}}\left(0\right)\right)\exp\left\{-\frac{2AP}{V}t\right\}}{c\left(\infty \right)+0.5\left(c_{\mathrm{high}}\left(0\right)-c_{\mathrm{low}}\left(0\right)\right)\exp\left\{-\frac{2AP}{V}t\right\}}$$

These time dependences of concentrations and retention with time-decreasing exponentials allow us to speak of a characteristic time in the process, *t*_c_, defined by the logarithmic decrement. The values of the characteristic time are shown in Table 1. All of them are values greater than hundreds of hours. The equilibrium in the system can be estimated to reach several times this amount. The measurements carried out in this work extended until 30 h, in either case—a situation still far from equilibrium. Therefore, it is important that referring to our measurements as “initial times” and “final times” has nothing to do with actual equilibrium.

The retention values were determined for the same instants of time as the permeability measurements, in two “initial” and “final times” periods, throughout the first 30 h of system evolution.

### 3.2. Electrochemical Impedance Spectroscopy

As already mentioned, EIS experiments for electrical characterization of solutions inside the pores of a membrane are usually performed by making a fixed concentration solution pass through the filtration membrane until equilibrium is reached [14,24], which is enough to give the electrical information of the system. In our case, as we wanted to study the dependence between the observed retention and the electrical properties of the membrane + solution, performing EIS measurements in equilibrium by using a fixed concentration, might not give the required information.

Since, in this work, we want to relate the results directly obtained from the EIS measurements to the retention and permeation properties of the membrane, the situation to be studied must be a nonequilibrium one; hence, there must be a concentration gradient between both sides of the membrane.

Given that the impedance measurements require a few minutes to obtain a complete spectrum of sufficiently representative frequencies, this study could not be carried out on membranes with a very short characteristic time of the order of seconds, as was previously shown [14]. In this case, the NF membranes give a slow enough evolution towards equilibrium with a sufficiently long characteristic time (Table 1).

Several measurements of the impedance spectra of the system were conducted at different instants. As mentioned, only the results at 0, 6, 23, and 29 h were used for representation and analysis in our graphs to allow an easy visualization. 

**Figure 4:**
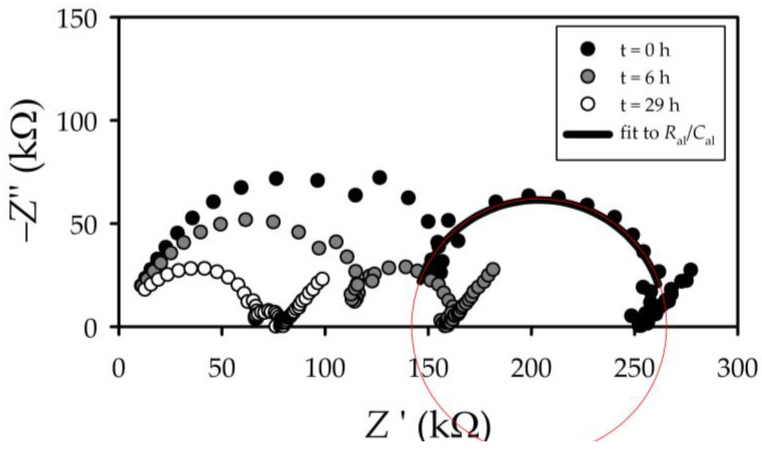
Nyquist diagrams for HL membrane and NaCl solution for different times (0, 6 and 29 h).

Figure 4 shows a typical example, for NaCl (the rest of the salts presented similar behaviors), of the temporal evolution of the impedance spectra. The figure shows the time evolution of the Nyquist diagram (imaginary impedance as a function of its real part) for a high starting concentration of 8.3×10^−3^ mol/L and a low concentration of 1.8×10^−5^ mol/L. EIS curves for three instants are shown. The high concentration barely changed from 8.3 to 7.8×10^−3^ mol/L. In the same period, on the other side of the membrane, the low concentration changed from 1.8×10^−5^ mol/L to 7.8×10^−5^ mol/L.

**Figure 5:**
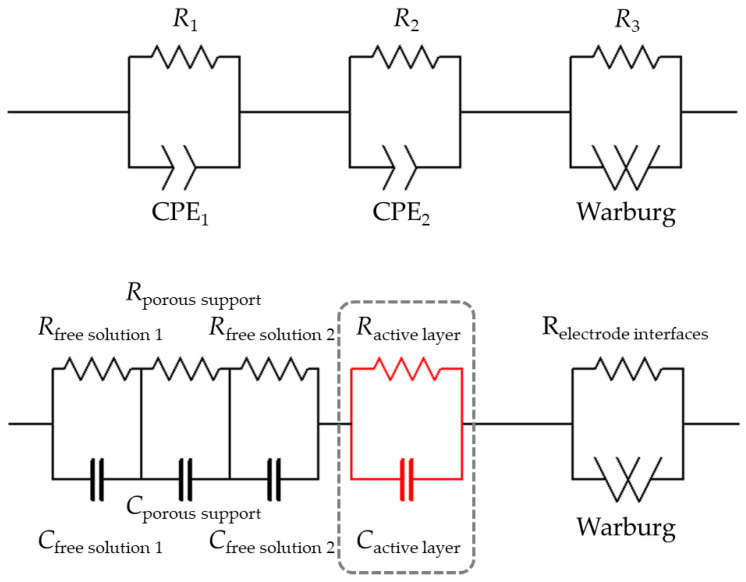
Equivalent electrical circuit for a system of an electrolyte solution inside the pores of a nanofiltration membrane.

The impedances displayed by the system for each frequency can be grouped into three lobes. At very high frequencies, the low real impedance corresponds to the first lobe. It relates to the behavior of the solution inside the support layer of the membrane and outside it. All these interactions, indistinguishable in the Nyquist plot, come together in a CPE1/R1 lobe in Figure 5. For very low frequencies, an incomplete lobe appears; for the highest real impedances, that corresponds to the relaxation of the polarization layer in contact with the electrodes in response to the applied field [24] (W/R3 in Figure 5).

The central lobe, corresponding to intermediate frequencies, will be the focus of interest of our study. This is because it is the lobe corresponding to the more restrictive part of the membrane, with the pores of the active layer of the membrane. The solution inside these pores clearly behaves differently than the solution outside them. 

The interpretation of these experimental EIS results needs to assume an electrical equivalent circuit, which is the common way for these measurement techniques [17,18]. It is presumed that the elements of a relatively simple electrical circuit can explain the data obtained by attending to their physical meaning in relation to the system under study. We chose the simplest model that could describe the system. More complex equivalent circuits could lead to a possible loss of the physical meaning of the parameters that describe each element.

For this case, the whole system formed by a NF membrane submerged in an electrolyte solution and measured using selective electrodes can be associated with an electrical circuit, as shown in Figure 5. The model consists of assuming that the support and the active layer are in a series association, each formed of a resistive element in parallel with a constant-phase element, and the solution in contact with the electrodes behaves as a resistive element in parallel with a Warburg element. Other authors [19] have explained in full detail the physical meaning of assuming such models, which we will use for the analysis of our data. In our case under study, the data corresponding to the effect of the Warburg element was ignored since they do not provide physical information about the NF membrane. In the same way, the part of the circuit corresponding to free dissolution was not determined either.

The experimental data interval corresponding to the central lobe was fitted to a single set in parallel. Since the lobe presents a shape reasonably close to a semicircle (see Figure 4), it was accepted as described by a parallel arrangement of a resistor (*R*_al_) and a pure capacity (*C*_al_), corresponding to the active layer. This electrical circuit, denoted here as *R*_al_/*C*_al_, is shown in red in Figure 5 inside the gray dashed rectangle. 

Under the assumption of the mentioned model with our simplifications, we can easily obtain the modelized impedance equation for each layer of the system membrane + solution by applying the laws of electrical circuits to the union of elements mentioned, which leads to a theoretical impedance for the support and active layer equal to Equation (6).

(6) $$Z_{\mathrm{al}}=\frac{R_{\mathrm{al}}\left[1-iR_{\mathrm{al}}C_{\mathrm{al}}\omega \right]}{1+R_{\mathrm{al}}{}^{2}C_{\mathrm{al}}{}^{2}\omega^{2}}$$

The experimental data for the central lobe can be fitted to Equation (6), which corresponds to the active layer response. In our case, we could observe that a good fitting can be obtained for all the experimental data and the proposed model.

**Figure 6:**
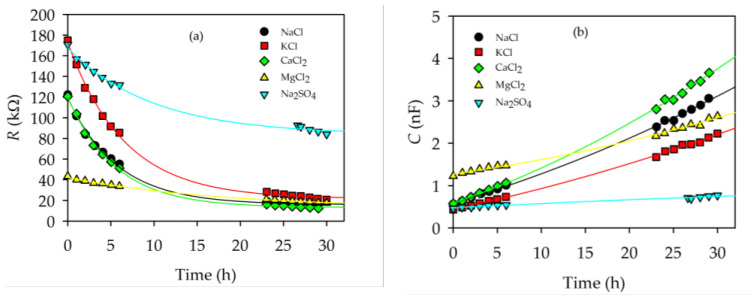
Time evolution of resistance (**a**) and capacitance (**b**) of the active layer of the studied NF membrane.

The mentioned fit of experimental values to the complex impedance of Equation (6) was performed for all the electrolyte solutions at different times. As can be seen in Figure 6, there was a time gap without measurements. Since the behavior of the system was sufficiently slow and smooth, the measurements were focused on the “initial” and “final” moments of the studied process, with the behavior of each one being easily interpolated during the period of nonexistence of measurements. The variation in the electrical resistance and capacitance obtained for the different solutions inside the active layer of the membrane is shown in Figure 6 for all the salt solutions used in this experiment.

Resistance decreased with time in all cases, whereas the capacity increased, as shown in Figure 6. The same experimental behaviour was observed in previous works [14]. Analyzing the time evolution, according to our division into “initial times” (≤6 h) and “final times” (≥23 h), a similar evolution in time was observed for all the studied saline solutions.

If we study the behavior of the active layer resistance, *R*_al_, the MgCl_2_ solution behaved somewhat differently from the rest. At “initial times”, its resistance values began by being considerably lower than those shown by the rest of the salts.

For “final times”, the solutions established in similar values for all the salts, except for Na_2_SO_4_. The still-negative slope after 30 h for *R*_al_ (Na_2_SO_4_), as well as its very long characteristic time (see Table 1), would indicate that the system was still very far from reaching equilibrium. In any case, this behavior is expected for this salt since, as we will see later, both from the electrical and steric point of view, equilibrium is unfavorable for the entry of this salt into the pores.

The value of *C*_al_ grew from an almost equal value for all solutions to a value for “final times” that showed the following behavior: *C*_al_(CaCl_2_) > *C*_al_(NaCl) > *C*_al_(KCl) > *C*_al_(Na_2_SO_4_). Only the MgCl_2_ solution presented a slightly different behavior for the value of *C*_al_: firstly, because the value shown at the initial time was somewhat higher than that of the other electrolytic solutions, and secondly, because its temporal evolution had a smoother trend than for the rest of the solutions. This gentle trend finally caused the value of parameter *C*_al_ for the “final times” to end up below that of NaCl and CaCl_2_.

For MgCl_2_, it is convenient to note that Mg^2+^ is the smallest ion (see Table 2), which would allow a greater mobility inside the pores and, thus, a low initial resistance and a relatively high initial capacity. In both cases, NaCl and CaCl_2_ gave more outstandingly low resistances and high capacities for the final stages.

### 3.3. Zeta Potential

The zeta potential of our NF membrane for each one of the electrolyte solutions was calculated using the method of tangential streaming potential. In this technique, the gradient pressure (Δ*P*) between both extremes of the single channel drives to a gradient of electric potential (Δ*U*); both magnitudes are measured. Assuming that there is a nonzero conductivity only in the section of the channel where the electrolyte solution is flowing, the streaming potential coefficient, (Δ*U*/Δ*P)_I_*_=0_, is related to zeta potential (ζ) with the Helmholtz–Smoluchowski equation [33,34,35]:

(7) $$\zeta =\frac{\eta \kappa }{\varepsilon_{r}\varepsilon_{0}}{\left(\frac{\Delta U}{\Delta P}\right)}_{I=0}$$

where η is the dynamic viscosity, κ is the solution conductivity, and $\varepsilon_{r}\varepsilon_{0}$ the permittivity of the solution. Under the assumption of greatly diluted solutions, the dynamic viscosity and the electrical permeability of the solution can be assumed to be equal to the values of pure water [36].

For each electrolyte solution, the streaming potential was measured, using solute concentrations close to the value of the feed used for EIS measurements. Data corresponding to the streaming and the zeta potentials are shown in Table 3.

### 3.4. Zeta Potential and Permeability

**Figure 7:**
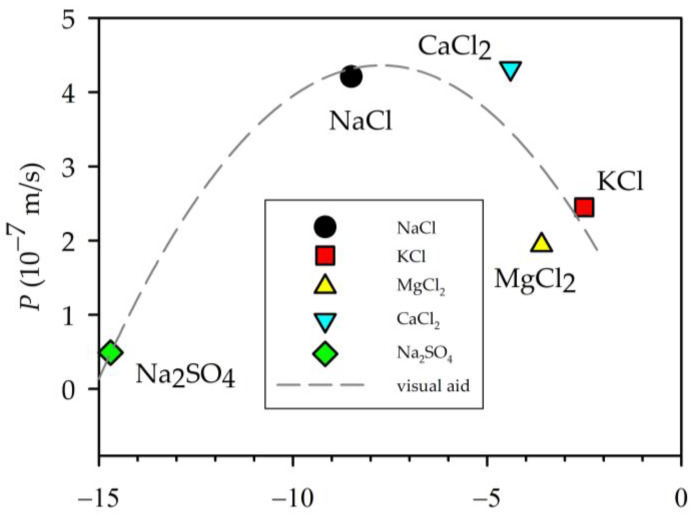
Dependence between zeta potential values and the permeability. The parabolic line shown is only an eye guide.

As we can see, from the zeta potential measurements, our membrane showed a negative charge on the surface for all the solutions used, which implies that a retention of anions was produced at the membrane. In our case, chlorine or sulphate was retained, while the cations were attracted by the membrane. Figure 7 shows the experimental permeability as a function of the zeta potential. The permeability seemed to decline with a decrease in the negative charge of the membrane, except for Na_2_SO_4_, that, as mentioned, would not have reached equilibrium; anyway, it is quite difficult for this salt to enter the pores.

### 3.5. Zeta Potential and EIS Parameters

Zeta potential has been observed to have a relation with conductivity values inside the pores of UF membranes [22], and a similar correlation should appear for NF membranes.

**Figure 8:**
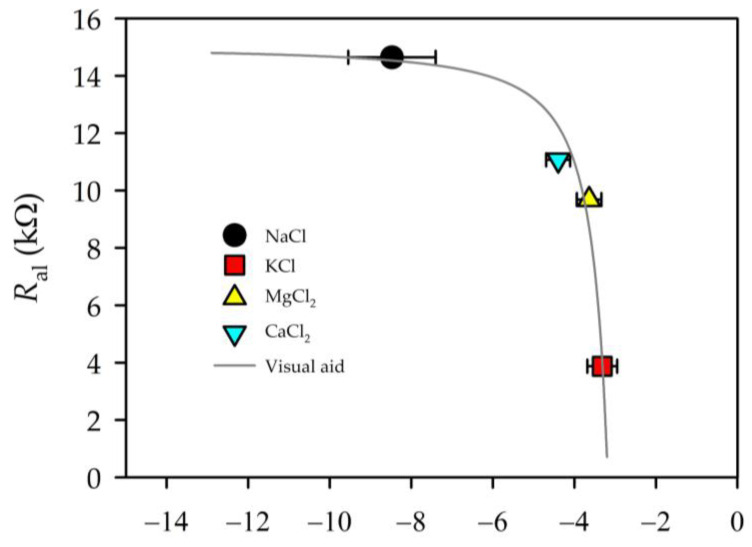
Dependence between zeta potential values and the resistance (calculated for the case of a charge concentration difference of 7×10^−4^ mol/L between both sides of the membrane).

In Figure 8, we can observe that there was a certain relation between the membrane resistance and the value of the zeta potential. This relation of the active layer resistance of the membrane was similar to that shown by Fievet et al. [22] for the case of UF membranes.

It is worth noting that *R*_al_ declined when the concentration of ions inside the pores decreased. Of course, *C*_al_ must and did show an opposite tendency. The entrance of ions into the pores must be controlled by the balance of ions outside and inside the active layer of the membrane:

(8) $$\frac{c_{j}}{{\bar{c}}_{j}}=\phi_{j}\frac{{\bar{\gamma }}_{j}}{\gamma_{j}}\exp\left(-q_{j}\Delta \Psi \right)\exp\left(-\Delta W_{j,\mathrm{Born}}^{’}\right)\exp\left(-\Delta W_{j,\mathrm{im}}^{’}\right)$$

**Figure 9:**
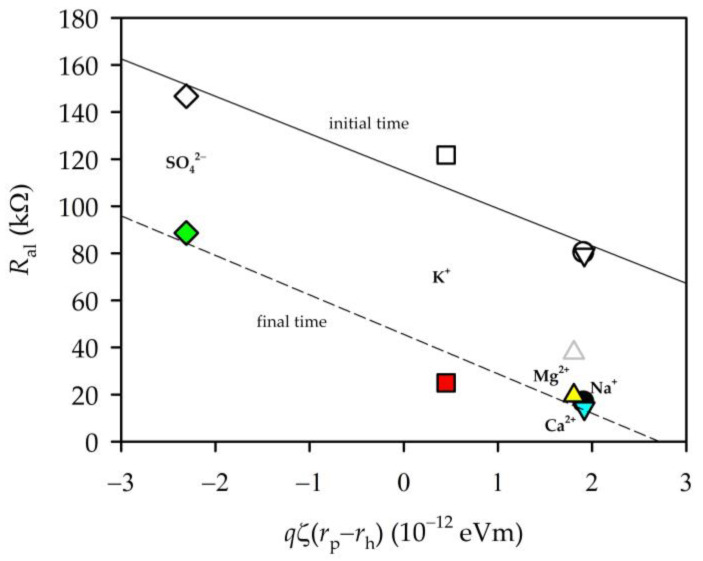
Variation in resistance as a function of the product [$q\zeta \left(r_{p}-r_{h}\right)$].

Here, ${\bar{c}}_{j}$ stands for the concentration inside the membrane, and $c_{j}$ for the concentration outside the membrane; ${\bar{\gamma }}_{j}$ and $\gamma_{j}$ are the corresponding activity coefficients; $\phi_{j}$ takes into account the steric effect; and ΔΨ is the normalized Donnan potential. Dielectric effects consist of the Born contribution, $\Delta W_{j,\mathrm{Born}}^{’}$, and those of image forces, $\Delta W_{j,\mathrm{im}}^{’}$ [19,31,37]. In other words, $c_{j}/{\bar{c}}_{j}$ will depend on three factors: the size relationship between the pore and the ions; the electrical potential of the membrane (magnitude and sign); and the dielectric effects. Without going into the quantification of these magnitudes, the access of an ion into a pore will be energetically favourable when its size is much smaller than that of the pore. On the other hand, it will also depend on the charge of the ion with respect to that of the membrane. In both cases, this is important for both ions since the condition of electroneutrality has to be maintained. With these considerations, we analysed how the resistance behaves as a function of the product of the elemental charge of the ion (*q*) times the zeta potential (which will account for the charge of the membrane) and multiplied by the difference between the pore radius and the radii of the hydrated ion ($r_{p}-r_{h}$), taking the latter as the mean ion–water intermolecular distance [36]. See Table 2 for ionic radii; it should be taken into account that, in a previous work, the pore radius of the membrane studied here was shown to be 0.46 nm [19]. We did not consider the chloride ion since it is common to all salts (except in the case of sulphate) and its concentration is the same in all cases. Figure 9 shows the behaviour of *R*_al_ for the average value of the “initial” and “final times” of each of the salts.

Figure 9 clearly shows a linear decrease in resistance for “final times”. For “initial steps”, a similar trend was followed. The behaviour of the capacities was similar, but in this case increasing.

This observation could help to explain the obtained permeability measurements, in the sense that zeta potential was correlated with retention [10,11]. A higher retention would imply a decrease in the diffusivity inside the pores, as a reduction in the effective pore radii was produced and the ions had difficulties in passing through the membrane (see Table 2). Ionic radii will have an important role in the effect of the value of permeability, as higher ionic radii will mean a lower permeability value. The combination of these effects will have produced the observed values for permeability.

### 3.6. Permeability and EIS Parameters

**Figure 10:**
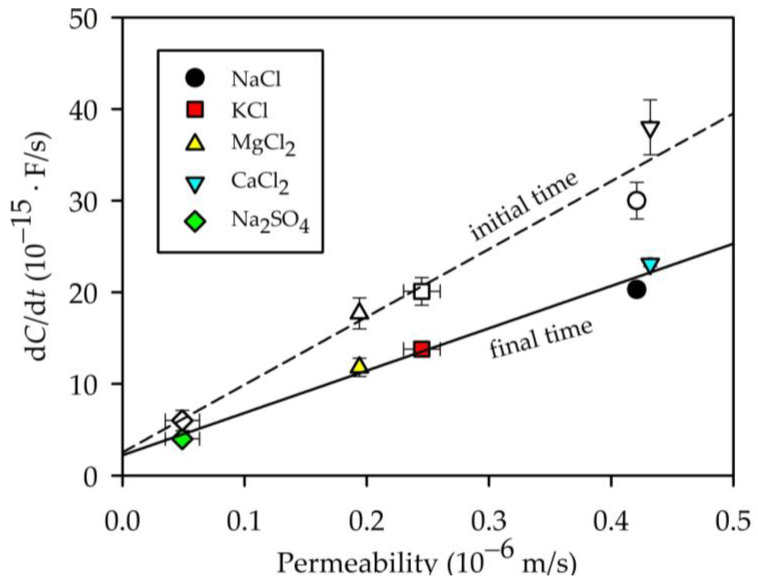
Dependence between salt permeability and the variation in the capacity with time inside the pores of the active layer of the membrane at “initial” and “final times” of the experiment.

Permeabilities are correlated with the time rate of change of capacitance, as seen in Figure 10, where it is shown that there were fair linear correlations for both initial and final steps.

It should be considered that permeability will be conditioned by the energetic jump of the ions from the solution to the pore, and also by their movement inside the pore. A clear correlation between permeability and diffusivity was not found, which means that once the ions entered the pore, they were also affected by the electric field created by adsorbed charge and by steric effects.

**Figure 11:**
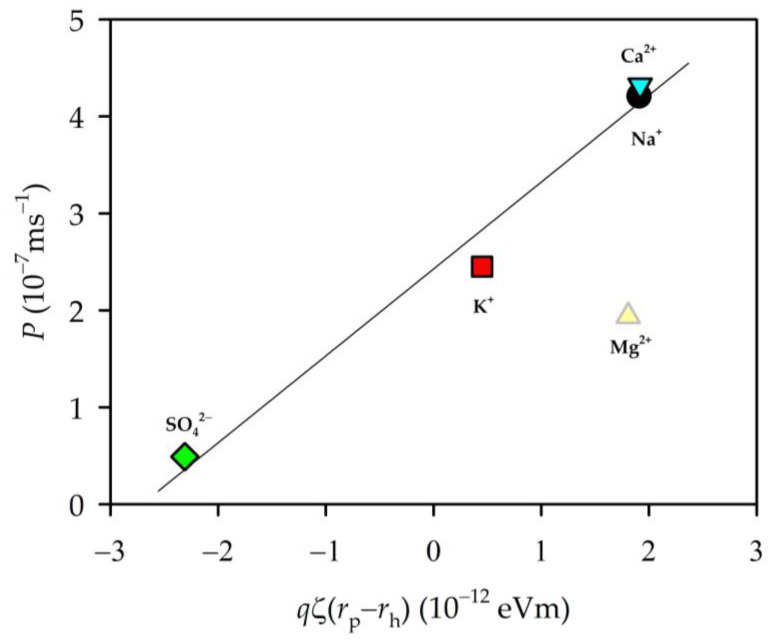
Variation in permeability as a function of the product [$q\zeta \left(r_{p}-r_{h}\right)$].

Similar to Figure 9, if we represent the permeability as a function of the product [$q\zeta \left(r_{p}-r_{h}\right)$] (see Figure 11), we see a linear behaviour for all the ions except magnesium chloride. This must be slowed down inside the pore by the effects of a strong electric field due to its high concentration inside the pore, as shown by the resistance values. If permeability is plotted against electrical resistance or capacitance, a similar (linear) behaviour is observed where, again, magnesium chloride is out of that trend (see Figure 9).

**Figure 12:**
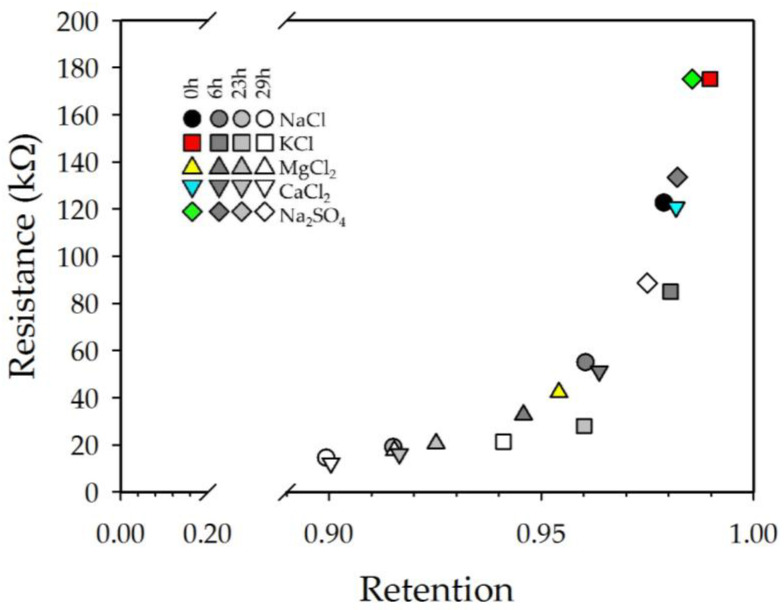
Dependence between the electrical resistance of the active layer of the membrane for each electrolyte solution at different stages of the experiment: 0 h, 6 h, 23 h, and 29 h.

A plot of the resistance at different times against the value of retention at that moment is shown in Figure 12 for four different stages of the experiment (t = 0, 6, 23, and 29 h).

We can observe that during the first hours of the beginning of the experiment (“initial times”), there was no clear relationship between the retention and the resistive behavior of the membrane, which can be explained by the fact that at the beginning of the experiment, a stationary equilibrium had not be reached inside the pores of the membrane. Meanwhile, after 6 h had passed, a relationship between both parameters could be observed, with this being clearer after 29 h. This phenomenon can be explained as the reach of an equilibrium inside the pores of our membrane, allowing for the observation that the resistive behavior of the membrane is correlated with the retention at that time following an exponential-like relation.

## 4. Conclusions

Different permeation and electrical parameters were measured, finding a correlation between them for a NF membrane when an electrolyte solution passed through it. This analysis included the retention to the passing of certain salts.

The resistance and capacitance values obtained from the EIS measurements had an opposite time evolution. Due to the increase in charges in movement inside the pores, membrane capacitance increased with time, whereas resistance decreased.

Zeta potential allowed us to know that our membrane was negative charge, which determined the movement of the ions inside the pores. Analyzing the behavior of resistance and permeability with a product that includes the charge of the membrane, pore radii, and the radii of the hydrated ion, we detected a difference between Mg^2+^ and the rest of the ions, an anomaly already detected in the time evolution of the EIS parameters.

The time variation in capacitance depended on salt permeability for all the salt solutions analyzed, with a different behavior in the first stage of the process (“initial times”) and the “final times”.

Additionally, a correlation between resistance and retention was observed. This dependence accounted for the nonequilibrium situation at the “initial times”, and the equilibrium reached inside the pores at the end of the process.

For the property of retention, we also observed an exponential-like dependence between both mentioned parameters, which can be explained by the phenomenon that when a certain molecule is highly retained inside the pores of a membrane, it will present a high resistance to the passing of an electrical signal, which was observed in our measurements. For the zeta potential dependence, a similar relation was obtained by Fievet et al. [22] between the conductivity of a solution inside the pores of a UF membrane and the value of zeta potential. In our study, we measured the electrical resistance of the active layer of a NF membrane, which was proportional to the value of conductivity, implying that the similar relation we observed should be obtained. Lastly, we obtained a relationship between the permeability value and the capacitance variation with time, which an active layer presents when a solution is passing through it.

The observed dependence between electrical parameters obtained through EIS and retention properties show the potential that this technique may have in the field of membrane characterization, as it would not just give certain parameters with precision, but it could allow reducing the experimental setups and time needed for the characterization of NF membranes. The correlation we found allows us to measure electrical parameters and foresee permeability and retention values.

## Figures and Tables

**Table 1:** Permeability obtained through a linear fit according to Equation (2) and the characteristic time (*t*_c_).

Electrolyte Solution	Permeability (10^−7^ m s^−1^)	*t*_c_ (h)
NaCl	4.21 ± 0.02	178
KCl	2.45 ± 0.15	315
MgCl_2_	1.94 ± 0.01	385
CaCl_2_	4.32 ± 0.01	175
Na_2_SO_4_	0.49 ± 0.14	1378

**Table 2:** Ionic radius, hydrated radius, and infinite dilution diffusivity, from the literature, for the ions studied in this work.

Ion	Ionic Radius (nm) [30]	Hydrated Radius, *r*_h._ (nm) [31]	Ionic Diffusivity (10^−5^ cm^2^ s^−1^) [32]
Na^+^	0.098 ± 0.003	0.2356 ± 0.0060	1.334
K^+^	0.134 ± 0.004	0.2798 ± 0.0081	1.957
Mg^2+^	0.072 ± 0.002	0.2090 ± 0.0041	0.706
Ca^2+^	0.103 ± 0.003	0.2422 ± 0.0052	0.792
SO_4_^2−^	0.240 ± 0.005	0.3815 ± 0.0071	1.065
Cl^—^	0.183 ± 0.003	0.3187 ± 0.0067	2.032

**Table 3:** Streaming potential and zeta potential for the membrane in contact with the studied electrolytes.

Electrolyte Solution	Streaming Potential (µV Pa^−1^)	Zeta Potential (mV)
NaCl	−0.55 ± 0.07	−8.5 ± 1.1
KCl	−0.16 ± 0.02	−2.5 ± 0.4
MgCl_2_	−0.18 ± 0.02	−3.6 ± 0.3
CaCl_2_	−0.20 ± 0.02	−4.4 ± 0.3
Na_2_SO_4_	−0.83 ± 0.03	−14.7 ± 0.4

## Data Availability

The data presented in this study are available on request from the corresponding author. The data are not publicly available due to privacy.
